# The risk of COVID-19 in survivors of domestic violence and abuse

**DOI:** 10.1186/s12916-021-02119-w

**Published:** 2021-09-24

**Authors:** Joht Singh Chandan, Anuradhaa Subramanian, Jaidev Kaur Chandan, Krishna M. Gokhale, Alecs Vitoc, Julie Taylor, Caroline Bradbury-Jones, Siddhartha Bandyopadhyay, Krishnarajah Nirantharakumar

**Affiliations:** 1 Institute of Applied Health Research, College of Medical and Dental Sciences, University of Birmingham, Birmingham, B152TT UK; 2 Warwick Medical School, University of Warwick, Coventry, CV47HL UK; 3 School of Nursing, College of Medical and Dental Sciences, University of Birmingham, Birmingham, B152TT UK; 4 Birmingham Women’s and Children’s Hospitals NHS Foundation Trust, Birmingham, UK; 5 The Department of Economics, University of Birmingham, Birmingham, B152TT UK

**Keywords:** Domestic abuse, Domestic violence, Epidemiology, COVID-19

## Abstract

A ‘shadow pandemic’ of domestic violence and abuse (DVA) has emerged secondary to strict public health measures containing the spread of SARS-CoV-2. Many countries have implemented policies to allow the free movement of DVA survivors in attempts to minimise their exposure to abusive environments. Although these policies are well received, as a result there is a possibility of increased COVID-19 transmission within this vulnerable group who are not currently prioritised for vaccination. Therefore, we aimed to compare the risk of developing suspected or confirmed COVID-19 in women (aged over 16 years) exposed to DVA against age-sex-matched unexposed controls, following adjustment for known COVID-19 risk factors. A population-based retrospective open cohort study was undertaken between the 31 January 2020 and 28 February 2021 using ‘The Health Improvement Network’ database. We identified 10,462 eligible women exposed to DVA who were matched to 41,467 similarly aged unexposed women. Following adjustment for key covariates, women exposed to DVA were at an increased risk (aHR 1.57; 95% CI 1.29–1.90) of suspected/confirmed COVID-19 compared to unexposed women. These findings support previous calls for positive policy action improving DVA surveillance and prioritising survivors for COVID-19 vaccination.

**Supplementary Information:**

The online version contains supplementary material available at 10.1186/s12916-021-02119-w.

## Background

A ‘shadow pandemic’ of domestic violence and abuse (DVA) has emerged secondary to strict public health measures containing the spread of SARS-CoV-2 [1]. Some of the risk factors mediating this relationship include movement restrictions, loss of income, isolation, overcrowding and high levels of stress and anxiety, all of which put victims of domestic abuse at a disproportionally increased risk of harm [2, 3]. The relationship between containment measures and increased rates of violence against women have occurred in other recent epidemics such as the Ebola outbreak in West Africa, Cholera outbreaks in Haiti and Yemen as well as the Zika outbreak [1, 4, 5]. Consequently, many countries have implemented policies to allow the free movement of DVA survivors in attempts to minimise their exposure to abusive environments and to facilitate access to support [6]. However, the encouraged freedom of travel (compounded by increased prevalence of comorbidities in DVA survivors) can potentially increase risk of COVID-19 exposure (and potential transmission) for this vulnerable cohort [7, 8]. To further exacerbate this risk, perpetrators may control or coerce survivors by (1) acting as a barrier preventing access to healthcare services or treatment or (2) threatening or enacting purposeful exposure to COVID-19 within the household [9, 10].

## Methods

We conducted a population-based retrospective open cohort study between the 31 January 2020 and 28 February 2021 using ‘The Health Improvement Network’ (THIN) database.

THIN database, hosted by Cegedim Health Data, consists of a sample of UK electronic medical records taken from 867 general practices (covering approximately 6% of the population) which have commissioned the use of the Vision software system [11, 12]. General practitioners (GPs), within the UK, have the option to subscribe to any of the NHS registered system suppliers and only those who have selected the Vision system will contribute data to THIN research database [13].

Following inception, THIN has been shown to be representative of the UK population in terms of demographic structure and prevalence of key comorbidities [14]. Symptoms, examinations, and diagnoses in THIN are recorded using a hierarchical clinical coding system called Read codes [15]. Of note, the database has been used extensively to examine outcomes of women exposed to DVA [7, 16–19]. General practices were included 1 year following their (1) instalment of electronic practice records and (2) acceptable mortality recording date to meet quality standards [20]. During the study period, 344 general practices (7,026,841 patients) met this inclusion criteria. Of those eligible general practices, only patients aged over 16 years, coded as female and registered with their general practice for at least 12 months prior to study start date (to ensure adequate time for baseline data recording to take place), were included. This led to a total of 2,512,769 remaining eligible patients for inclusion in the study.

Women (aged 16+ years) in the exposed group were defined as those with clinically coded exposure to DVA (Additional file 1: Table S1) who were then age- and sex-matched to those without such a recorded code (unexposed group). The Read code selection process has been previously described in the literature [21]. Ascertainment of exposure to DVA included the presence of broad codes such as ‘14X8.00: Victim of domestic violence’ as well as more specific exposures to sub-types of DVA such as ‘14XD200: H/O domestic sexual abuse’.

The index date in the exposed group was the date of the first code relating to DVA exposure or when they became eligible to enter the study. To mitigate immortality time bias, the same index date was assigned to the corresponding unexposed patient. The follow-up period for each patient was from the index date until the exit date, defined as the earliest of either of the following: study end date, last date of data collection from a given general practice, date patient transferred from general practice, date of death or date the outcome of interest occurred. The outcome of interest was the presence of a GP recorded diagnosis of suspected or confirmed COVID-19. Covariates (sociodemographic, lifestyle risk factors and comorbidities) relating to known risk factors for the development of COVID-19 complications were also recorded at each patient’s study entry [8].

Crude incidence rates per 1000 person-years were estimated for the exposed and the unexposed groups. A Cox proportional hazards regression model was then undertaken to determine crude and adjusted hazard ratios (HR) with 95% confidence intervals (CI) describing the COVID-19 risk in the exposed compared to the unexposed group. Where there were missing data in our covariates, they were treated as a separate missing category and included in the final analysis.

To examine the impact of recent DVA exposure and risk of outcome development, we have also undertaken a sensitivity analysis including only those with a code for DVA exposure in the 1 year prior to the study start date or during the study period.

## Findings

We identified 10,462 eligible women exposed to DVA who were matched to 41,467 similarly aged unexposed women. At study entry, in keeping with known literature [7], the exposed group had increased rates of smoking and a greater prevalence of comorbidities compared to the unexposed group (Table 1).

**Table 1:** Baseline characteristics

Total number	Exposed group	Unexposed group
	(***n***=10,462)	(***n***=41,467)
**Age [mean (SD)]**	42.38 (13.49)	41.74 (13.42)
**Age [median (IQR)]**	41.00 (32.00–51.00)	40.00 (32.00–50.00)
**Age categories [*n*(%)]**		
**16–30 years**	1797 (17.18)	7831 (18.88)
**30–40 years**	3087 (29.51)	12241 (29.52)
**40–50 years**	2561 (24.48)	10131 (24.43)
**50–60 years**	1914 (18.29)	7307 (17.62)
**60–70 years**	726 (6.94)	2610 (6.29)
**>70 years**	377 (3.60)	1347 (3.25)
**BMI [mean (SD)]**	27.09 (6.78)	26.47 (6.60)
**BMI [median (IQR)]**	25.00 (22.00-30.00)	24.00 (21.00-29.00)
**BMI categories [*n*(%)]**		
**Underweight (<18.5)**	433 (4.14)	1200 (2.89)
**Normal weight (18.5–24.9)**	3493 (33.39)	13913 (33.55)
**Overweight (25–30)**	2383 (22.78)	7362 (17.75)
**Obese (>30)**	2412 (23.05)	6970 (16.81)
**Missing**	1741 (16.64)	12022 (28.99)
**Smoker categories [*n*(%)]**		
**Non-smoker**	3690 (35.27)	19809 (47.77)
**Ex-smoker**	1807 (17.27)	5331 (12.86)
**Smoker**	4317 (41.26)	7989 (19.27)
**Missing**	648 (6.19)	8338 (20.11)
**Ethnicity [*n*(%)]**		
**Caucasian**	5780 (55.25)	13184 (31.79)
**South Asian**	456 (4.36)	785 (1.89)
**Black Afro-Caribbean**	303 (2.90)	510 (1.23)
**Mixed race**	117 (1.12)	225 (0.54)
**Chinese/Middle Eastern/others**	1848 (17.66)	6816 (16.44)
**Missing**	1958 (18.72)	19947 (48.10)
**eGFR [mean (SD)]**	99.98 (18.39)	100.04 (18.72)
**eGFR [median (IQR)]**	101.00 (88.00–112.00)	101.00 (87.00–113.00)
**eGFR categories [*n*(%)]**		
**Stage 1/2 (>60 mL/min)**	7020 (67.10)	17329 (41.79)
**Stage 3 (30–59 mL/min)**	135 (1.29)	293 (0.71)
**Stage 4 (<30 mL/min)**	8 (0.08)	33 (0.08)
**Missing**	3299 (31.53)	23812 (57.42)
**Systolic BP [mean (SD)]**	120.42 (15.23)	119.67 (14.55)
**Systolic BP [median (IQR)]**	120.00 (110.00–130.00)	120.00 (110.00–130.00)
**Systolic BP categories [*n*(%)]**		
**Normal (<120 mm Hg)**	4463 (42.66)	15294 (36.88)
**Prehypertension (120–129 mm Hg)**	2357 (22.53)	8803 (21.23)
**Hypertension stage 1 (130–139 mm Hg)**	1529 (14.61)	5159 (12.44)
**Hypertension stage 2 (140–149 mm Hg)**	1066 (10.19)	3003 (7.24)
**Missing**	1047 (10.01)	9208 (22.21)
**Other co-morbidities [*n*(%)]**		
**Hypertension**	2289 (21.88)	6591 (15.89)
**Diabetes**	449 (4.29)	881 (2.12)
**Cancer**	225 (2.15)	614 (1.48)
**Cardiac diseases**	373 (3.57)	627 (1.51)
**Myocardial infarction**	71 (0.68)	109 (0.26)
**Heart failure**	43 (0.41)	96 (0.23)
**Stroke/TIA**	205 (1.96)	274 (0.66)
**Atrial fibrillation**	40 (0.38)	79 (0.19)
**Peripheral vascular disease**	83 (0.79)	147 (0.35)
**Chronic liver disease**	190 (1.82)	167 (0.40)
**Mild liver disease**	185 (1.77)	163 (0.39)
**Moderate/severe liver disease**	22 (0.21)	14 (0.03)
**Neuron disease**	330 (3.15)	674 (1.63)
**Parkinson’s disease**	10 (0.10)	9 (0.02)
**Motor neuron disease**	1 (0.01)	1 (0.00)
**Multiple sclerosis**	35 (0.33)	92 (0.22)
**Epilepsy**	286 (2.73)	572 (1.38)
**Myasthenia gravis**	2 (0.02)	5 (0.01)
**Rheumatic diseases**	589 (5.63)	1455 (3.51)
**SLE**	30 (0.29)	38 (0.09)
**Rheumatoid arthritis**	82 (0.78)	150 (0.36)
**Psoriasis**	489 (4.67)	1283 (3.09)
**Dementia**	45 (0.43)	64 (0.15)
**Severe respiratory disease**	69 (0.66)	99 (0.24)
**Asthma**	2296 (21.95)	5781 (13.94)
**COPD**	310 (2.96)	296 (0.71)
**Solid organ transplant**	7 (0.07)	23 (0.06)
**Prescriptions (any time prior to index date) [*n*(%)]**		
**Immunosuppressive drug therapies**	25 (0.24)	70 (0.17)

During the study period, there were 190 (1.8%) new diagnoses of suspected/confirmed COVID-19 in the exposed cohort relating to an IR of 20.2 per 1000 person years compared to 8.6 per 1000 person years (324 (0.8%) in the unexposed cohort (Table 2). This translated to an unadjusted HR of 2.36 (95% CI 1.98–2.83). Following adjustment for key covariates (these include (1) sociodemographic characteristics—age and ethnicity; (2) lifestyle and metabolic profile measures—smoking status, body mass index (BMI), systolic and diastolic blood pressure and estimated glomerular filtration rate (eGFR); (3) comorbidity—type 2 diabetes mellitus, cardiovascular disease (peripheral vascular disease, stroke, ischaemic heart disease, atrial fibrillation and heart failure), severe respiratory disease, asthma, chronic obstructive pulmonary disease, cancer, liver disease (mild, moderate and severe), rheumatic disease (rheumatoid arthritis, lupus and psoriasis) and neurological disorders (Parkinson’s disease, motor neuron disease, multiple sclerosis, myasthenia gravis and epilepsy), dementia, solid organ transplants, and use of immunosuppressive drug therapies), the risk remained statistically significant (aHR 1.57; 95% CI 1.29–1.90). When examining suspected (aHR 1.61; 95% CI 1.27–2.03) and confirmed (aHR 1.56; 95% CI 1.13-2.16) COVID-19 outcomes separately, the findings remained similar.

**Table 2:** The risk of developing COVID-19 in the exposed group compared to the unexposed group

Total number of patients	Exposed group	Unexposed group
	10,462	41,467
**Suspected/confirmed COVID-19**		
**Outcome events [*n*** **(%)]**	190 (1.82)	324 (0.78)
**Person-years**	9406	37,901
**Crude incidence rate/1000 person years**	20.2	8.55
**Unadjusted hazard ratio (95% CI)**	2.36 (1.98–2.83), *p*<0.001	
**Adjusted hazard ratio (95% CI)**^**a**^	1.57 (1.29–1.90), *p*<0.001	
**Confirmed COVID-19**		
**Outcome events [*n*** **(%)]**	58 (0.55)	135 (0.33)
**Person-years**	9484	38,011
**Crude incidence rate/1000 person years**	6.12	3.55
**Unadjusted hazard ratio (95% CI)**	1.73 (1.27–2.35), *p*<0.001	
**Adjusted hazard ratio (95% CI)**^**a**^	1.56 (1.13–2.16), *p*=0.007	
**Suspected COVID-19**		
**Outcome events [*n*** **(%)]**	138 (1.32)	193 (0.47)
**Person-years**	9421	37,946
**Crude incidence rate/1000 person years**	14.65	5.09
**Unadjusted hazard ratio (95% CI)**	2.87 (2.31–3.57), *p*<0.001	
**Adjusted hazard ratio (95% CI)**^**a**^	1.61 (1.27–2.03), *p*<0.001	

^a^Adjusted for currently known risk factors for the development of COVID-19. These include (1) sociodemographic characteristics—age and ethnicity; (2) lifestyle and metabolic profile measures: smoking status, body mass index (BMI), systolic and diastolic blood pressure and estimated glomerular filtration rate (eGFR); (3) comorbidity—type 2 diabetes mellitus, cardiovascular disease (peripheral vascular disease, stroke, ischaemic heart disease, atrial fibrillation and heart failure), severe respiratory disease, asthma, chronic obstructive pulmonary disease, cancer, liver disease (mild, moderate and severe), rheumatic disease (rheumatoid arthritis, lupus and psoriasis), neurological disorders (Parkinson’s disease, motor neuron disease, multiple sclerosis, myasthenia gravis and epilepsy), dementia, solid organ transplants and use of immunosuppressive drug therapies

There were 1151 women exposed to DVA in the 1 year prior to the study start date or during the study period eligible in the sensitivity analysis who were matched to 4561 women. The results were largely similar to the main cohort whereby the exposed group had a further increased risk of a new diagnosis of suspected/confirmed COVID-19 following adjustment for key covariates (aHR 2.53; 95% CI 1.51–4.26), although it should be noted the confirmed case only subgroup analysis did not reach statistical significance. Further details can be seen in additional file 1: Table S2.

## Discussion

Our findings demonstrate that despite accounting for known risk factors, individuals exposed to DVA were at an increased risk of suspected/confirmed Covid-19 as recorded in general practitioner records. It is therefore possible that a clinical-safeguarding paradox may exist whereby current policy action protecting survivors from further abuse may indeed increase their risk of COVID-19. These findings support our previous calls for positive policy action improving DVA surveillance and prioritising survivors for COVID-19 vaccination [6, 22].

Although such positive action would be recommended, we also appreciate that this cohort are traditionally a hard-to-reach population often due to (1) controlling behaviours exerted by perpetrators, (2) the extent of the hidden burden of DVA not known to public sector services and (3) inadequate historical public sector responses to support this cohort leading to mistrust [23]. Additionally, practical and logistical challenges may be present for survivors further reducing their access to vaccine clinics. For example, survivors may not have access to transportation or the means to afford public travel due to ongoing financial abuse. Therefore, we recommend countries adopt the evidence-based recommendations suggested by Gavi to increase vaccination rates in hard-to-reach populations with an initial focus on reducing physical barriers to vaccine access [24]. Practically, these recommendations may include setting up pop-up clinics in the community minimising travel or where survivors have already been displaced, for example, to set up clinics within domestic violence shelters.

Despite this study having a large sample size which has been previously examined to assess health outcomes in the DVA population, the findings must be considered in light of the study limitations. Misclassification bias poses a key limitation in this study, as the prevalence of DVA in THIN (<0.4%) is substantially lower than the self-reported rates in the UK [25]. Although previous studies examining the health outcomes in this cohort have shown similar effect sizes to the published literature, DVA coding has yet to be validated in THIN [7, 16]. Despite the lack of validation specifically in this dataset, promisingly a recent meta-analysis demonstrated a high positive predictive value (>85%) of intimate partner violence in other electronic health records, providing some confidence in the accuracy of recorded DVA experiences [26]. Therefore, although we may not be able to truly determine whether the misclassification bias could lead to an over or under-estimate, it appears more likely that the unexposed cohort will contain a greater proportion of miscoded events (where DVA may have occurred but was not coded) rather than the exposed cohort having miscoded events whereby DVA occurred when in fact it did not. Therefore, if the hypothesis is correct that DVA exposure may lead to increased exposure to SARS-CoV-2 then we anticipate that some of the events in the unexposed cohort may have occurred in survivors exposed to DVA, which may in turn lead to reduction of our reported effect size. A recent review exploring DVA coding in healthcare identified that although primary care staff appreciate the importance of recording DVA, barriers still exist relating to lack of training, unawareness of local/national guidance, confidentiality concerns and how it may affect the patient-clinician relationship [27]. As no research to date has been published suggesting a difference in DVA severity or demographic characteristics between coded and uncoded experiences of DVA, it is not yet possible to identify whether there is a systematic difference between those in the exposed group and those miscoded in the unexposed group ultimately informing us as to how this may affect the effect size reported in this study. However, it does reaffirm the importance of ensuring that our results are used to describe the effect size in those with recorded exposure to DVA.

A second key limitation relates to the inability to account for the impact of socio-economic deprivation due to this data being unavailable for the cohort during the study period. However, to mitigate this as much as possible, we have been able to match patients by the location of the general practice. Although, heterogeneity in socio-economic status is likely to remain.

In conclusion, we believe this is the first study describing the risk of COVID-19 in DVA survivors and highlights the important global public health needs of one of the most vulnerable groups in society.

## Supplementary Information

**Additional file 1: Table S1.** Read codes used to describe exposure to domestic violence and abuse. **Table S2.** The risk of developing COVID-19 in the patients coded with DVA exposure in the one year prior to the study start date compared to the unexposed group

## Data Availability

The data and code for this study can be requested from the corresponding author and will be shared once approval is obtained from the data provider.

## Abbreviations

aHR: Adjusted hazard ratio
CI: Confidence interval
DVA: Domestic violence and abuse
HR: Hazard ratio
IR: Incidence rate
THIN: The Health Improvement Network
