# Supplementary material for: The risk of COVID-19 in survivors of domestic violence and abuse
Source: BMC Med. 2021 Sep 24;19:246. doi: 10.1186/s12916-021-02119-w (PMC8460316; doi:10.1186/s12916-021-02119-w)
Supplement: Supplementary file 1 — Additional file 1: Table S1. Read codes used to describe exposure to domestic violence and abuse. Table S2. The risk of developing COVID-19 in the patients coded with DVA exposure in the one year prior to the study start date compared to the unexposed group [file 12916_2021_2119_MOESM1_ESM.docx]

**Table S1: Read codes used to describe exposure to domestic violence and abuse**

| Read code | DESCRIPTION |
| --- | --- |
| 14X3.00 | History of domestic violence |
| 14X8.00 | Victim of domestic violence |
| 14XD.00 | History of domestic abuse |
| 14XD000 | H/O domestic emotional abuse |
| 14XD100 | H/O domestic physical abuse |
| 14XD200 | H/O domestic sexual abuse |
| 14XE.00 | History of being victim of domestic violence |
| 14XG.00 | Victim of domestic abuse |

**Table S2: The risk of developing COVID-19 in the patients coded with DVA exposure in the one year prior to the study start date compared to the unexposed group**

|  | **Exposed group** | **Unexposed group** |
| --- | --- | --- |
| **Total number of patients** | **1,151** | **4,561** |
| **Suspected/Confirmed COVID-19** |  |  |
| **Outcome events, [n (%)]** | 33 (2.87%) | 35 (0.77%) |
| **Person-years** | 951 | 3,899 |
| **Crude Incidence Rate/1000 person years** | 34.69 | 8.98 |
| **Unadjusted hazard ratio (95% CI)** | 3.86 (2.40-6.22) p<0.001 | |
| **Adjusted hazard ratio (95% CI)*** | 2.53 (1.51-4.26) p<0.001 | |
| **Confirmed COVID-19** |  |  |
| **Outcome events, [n (%)]** | 6 (0.52) | 15 (0.33) |
| **Person-years** | 965 | 3,908 |
| **Crude Incidence Rate/1000 person years** | 6.22 | 3.84 |
| **Unadjusted hazard ratio (95% CI)** | 1.63 (0.63-4.20) p=0.311 | |
| **Adjusted hazard ratio (95% CI)*** | 1.54 (0.56-4.25) p=0.408 | |
| **Suspected COVID-19** |  |  |
| **Outcome events, [n (%)]** | 28 (2.43) | 20 (0.44) |
| **Person-years** | 953 | 3,903 |
| **Crude Incidence Rate/1000 person years** | 29.39 | 5.12 |
| **Unadjusted hazard ratio (95% CI)** | 5.71 (3.22-10.13) p<0.001 | |
| **Adjusted hazard ratio (95% CI)*** | 3.05 (1.62-5.73) p<0.001 | |

* adjusted for currently known risk factors for the development of COVID-19. These include: 1) sociodemographic characteristics: age and ethnicity; 2) lifestyle and metabolic profile measures: smoking status, body mass index (BMI), systolic and diastolic blood pressure and estimated glomerular filtration rate (eGFR) 3) comorbidity: Type 2 diabetes mellitus, cardiovascular disease (peripheral vascular disease, stroke, ischaemic heart disease, atrial fibrillation and heart failure), severe respiratory disease, asthma, chronic obstructive pulmonary disease, cancer, liver disease (mild, moderate and severe), rheumatic disease (rheumatoid arthritis, lupus and psoriasis), neurological disorders (Parkinson’s disease, motor neuron disease, multiple sclerosis, myasthenia gravis and epilepsy), dementia, solid organ transplants, and use of immunosuppressive drug therapies.
